# Supplementary material for: Interplay between copper redox and transfer and support acidity and topology in low temperature NH3-SCR
Source: Nat Commun. 2023 May 6;14:2633. doi: 10.1038/s41467-023-38309-8 (PMC10164144; doi:10.1038/s41467-023-38309-8)
Supplement: Supplementary file 1 — Supplementary Information [file 41467_2023_38309_MOESM1_ESM.pdf]

Interplay between copper redox and transfer and support acidity and topology in low temperature NH_3-SCR

Yiqing Wu[1§], Wenru Zhao[2§], Sang Hyun Ahn[3], Yilin Wang[1], Eric D. Walter[1], Ying Chen[1], Miroslaw A. Derewinski[1, 4], Nancy M. Washton[1], Kenneth G. Rappé[1], Yong Wang[1, 5], Donghai Mei[2, 6*], Suk Bong Hong[3*], Feng Gao[1*]

1. Institute for Integrated Catalysis, Pacific Northwest National Laboratory, Richland, Washington 99354, United States;

2. School of Materials Science and Engineering, Tiangong University, Tianjin 300387, China;

3. Center for Ordered Nanoporous Materials Synthesis, School of Environmental Science and Engineering, POSTECH, Pohang 790-784, Republic of Korea;

4. J. Haber Institute of Catalysis and Surface Chemistry, Polish Academy of Sciences, 30-239 Krakow, Poland;

5. Voiland School of Chemical Engineering and Bioengineering, Washington State University, Pullman, Washington 99163, United States;

6. School of Environmental Science and Engineering, Tiangong University, Tianjin 300387, China.

§: these authors contributed equally to the work.

*: corresponding authors. Emails: dhmei@tiangong.edu.cn (D.H.M.), sbhong@postech.ac.kr (S.B.H.), feng.gao@pnnl.gov (F.G.)

Supplementary Figures and Tables

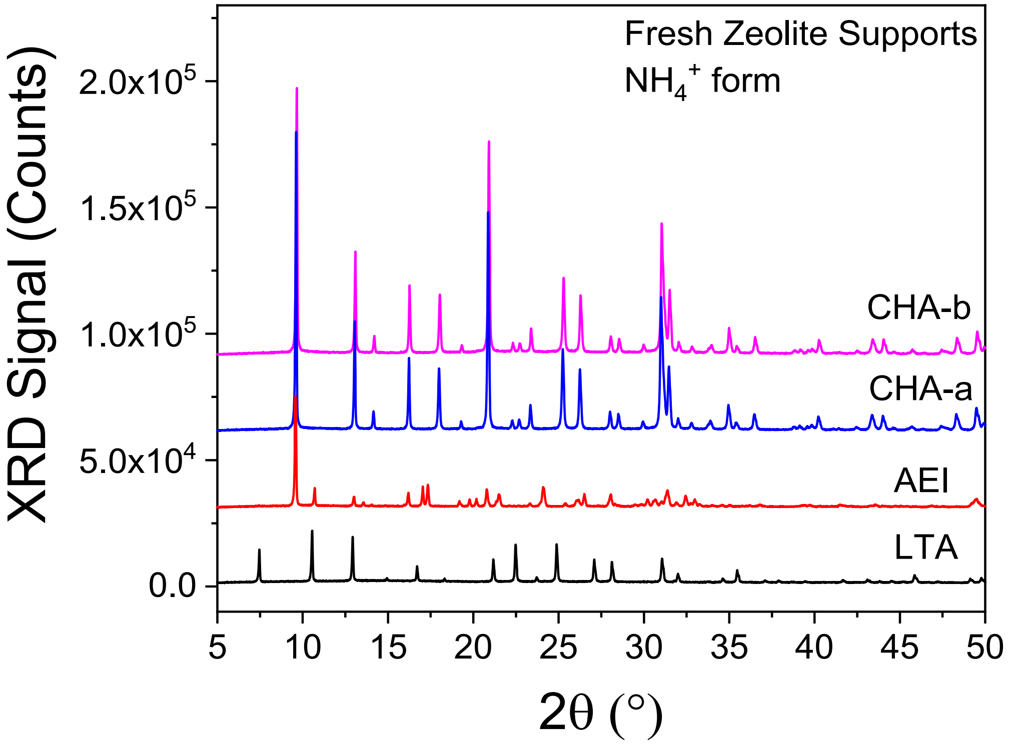

Supplementary Fig. 1 | PXRD patterns for the LTA, AEI and CHA-a/b supports in their NH_4-form. All supports display their pure phases without measurable impurity phases. The corresponding PXRD patterns for the fresh catalysts are highly similar and are thus not presented.

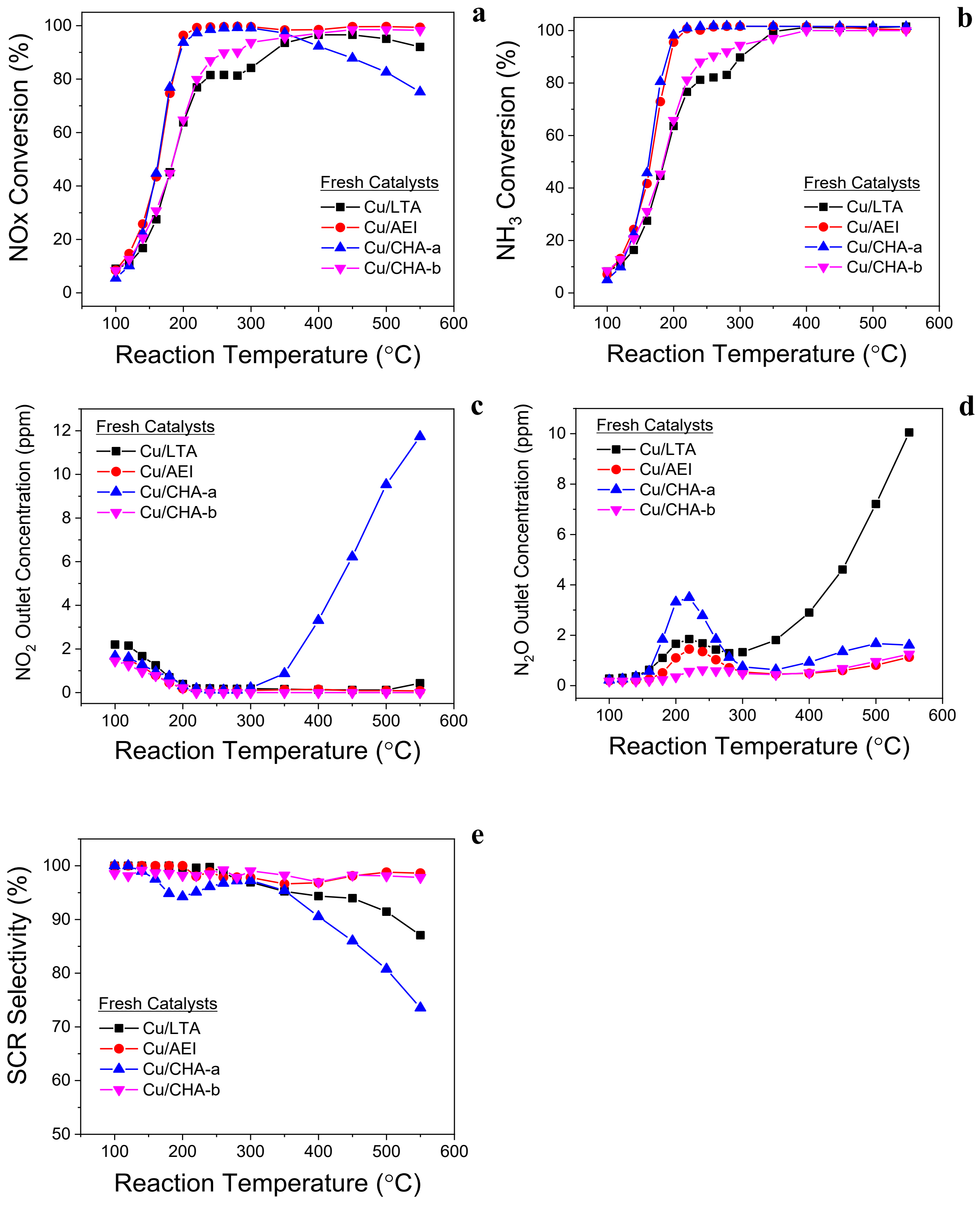

Supplementary Fig. 2 | Steady-state standard NH_3-SCR reaction test results on the 4 fresh catalysts. **a**, NOx conversion as a function of reaction temperature. **b**, NH_3 conversion as a function of reaction temperature. **c**, NO_2 outlet concentration (ppm) as a function of reaction temperature. **d**, N_2O outlet concentration (ppm) as a function of reaction temperature. **e**, SCR

selectivity as a function of reaction temperature. SCR selectivity is calculated as: $\left(\frac{NO_x\ conversion}{NH_3\ conversion} - \frac{N_2O\ outlet\ concentration}{NO_x\ inlet\ concentration}\right) \times 100\%$. The reactant feed contains 350 ppm NOx (including ~10 ppm NO_2), 350 ppm NH_3, 2.5% H_2O, 10% O_2, and balanced N_2 at a gas hourly space velocity (GHSV) of ~2×10^5 h^{-1}.

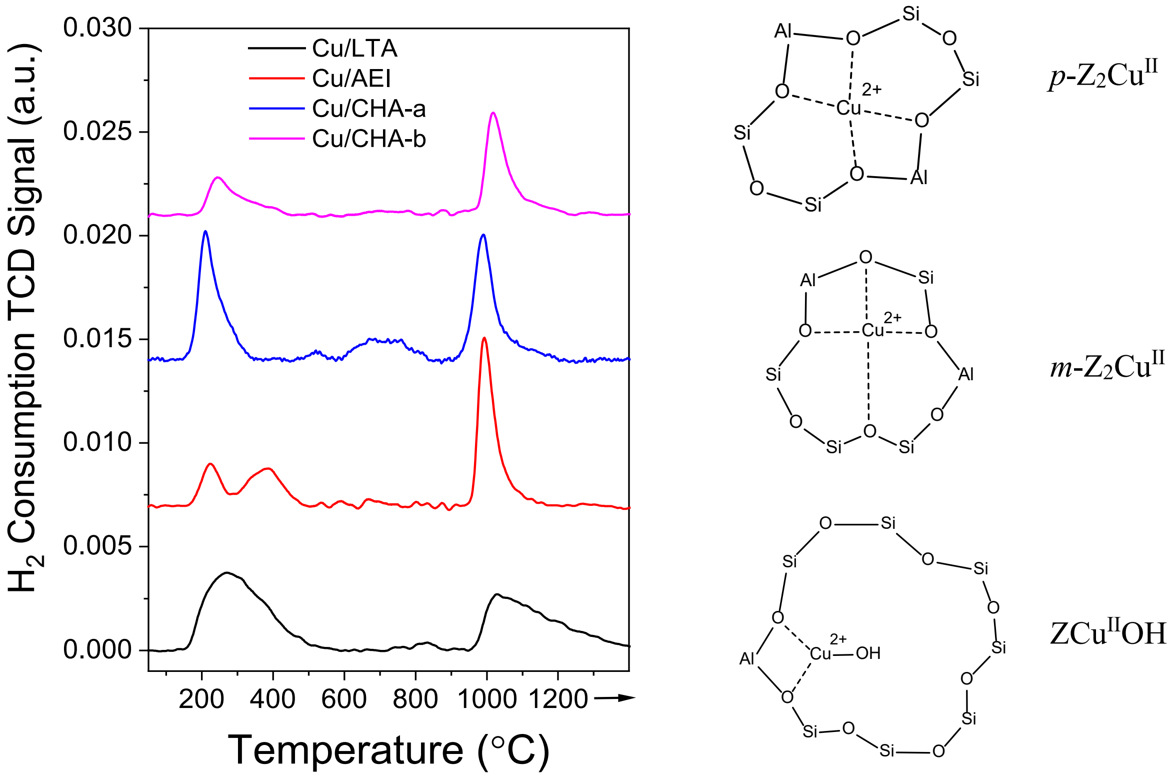

Supplementary Fig. 3 | H_2-TPR results for the 4 fresh catalysts. Reduction centered ~230 °C is due to $ZCu^{II}OH$ reduction to ZCu^{I}, reduction centered ~400 °C is due to Z_2Cu^{II} reduction to ZCu^{I}, and reduction above ~600 °C is due to ZCu^{I} reduction to metallic Cu[1, 2]. Schematics of Z_2Cu^{II} and $ZCu^{II}OH$ are shown on the right side of the TPR plots[3, 4].

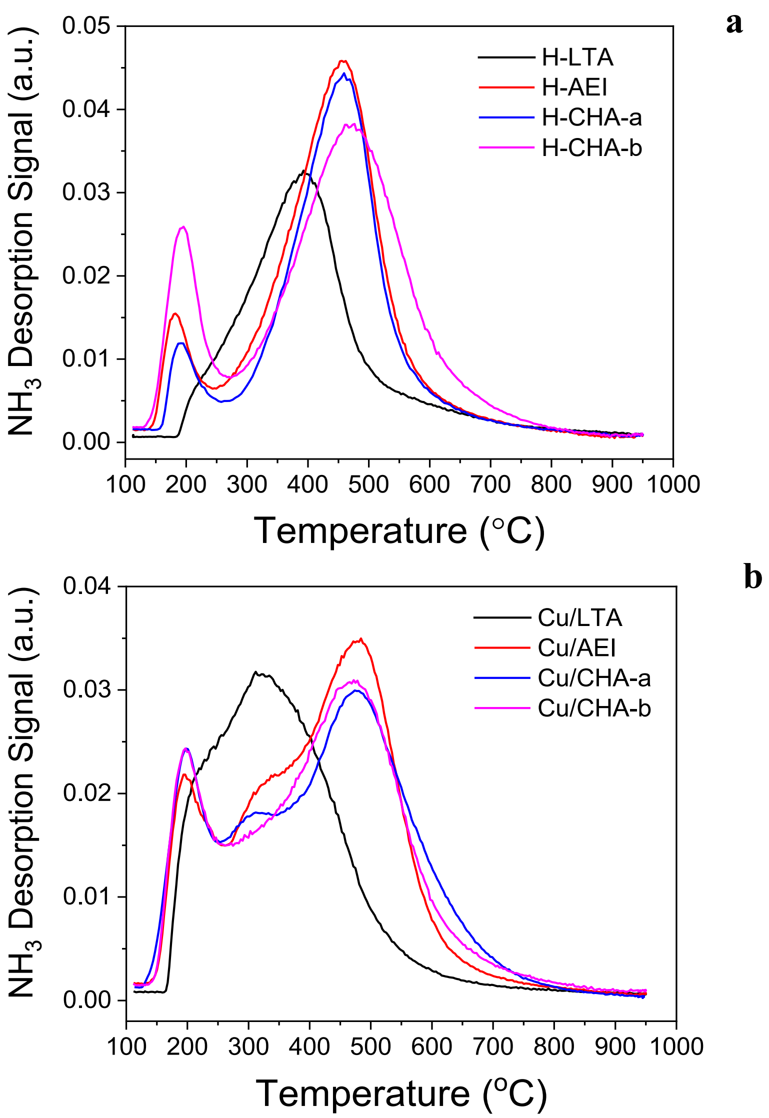

Supplementary Fig. 4 | NH_3-TPD results. a, H-form supports. **b**, fresh catalysts. Desorption from ~200 °C due to weak acid sites, ~300 °C due to Cu-ion sites, and ~400-500 °C to Brønsted acid sites[5]. The results demonstrate that AEI and CHA have similar Brønsted acid strength, higher than that of LTA.

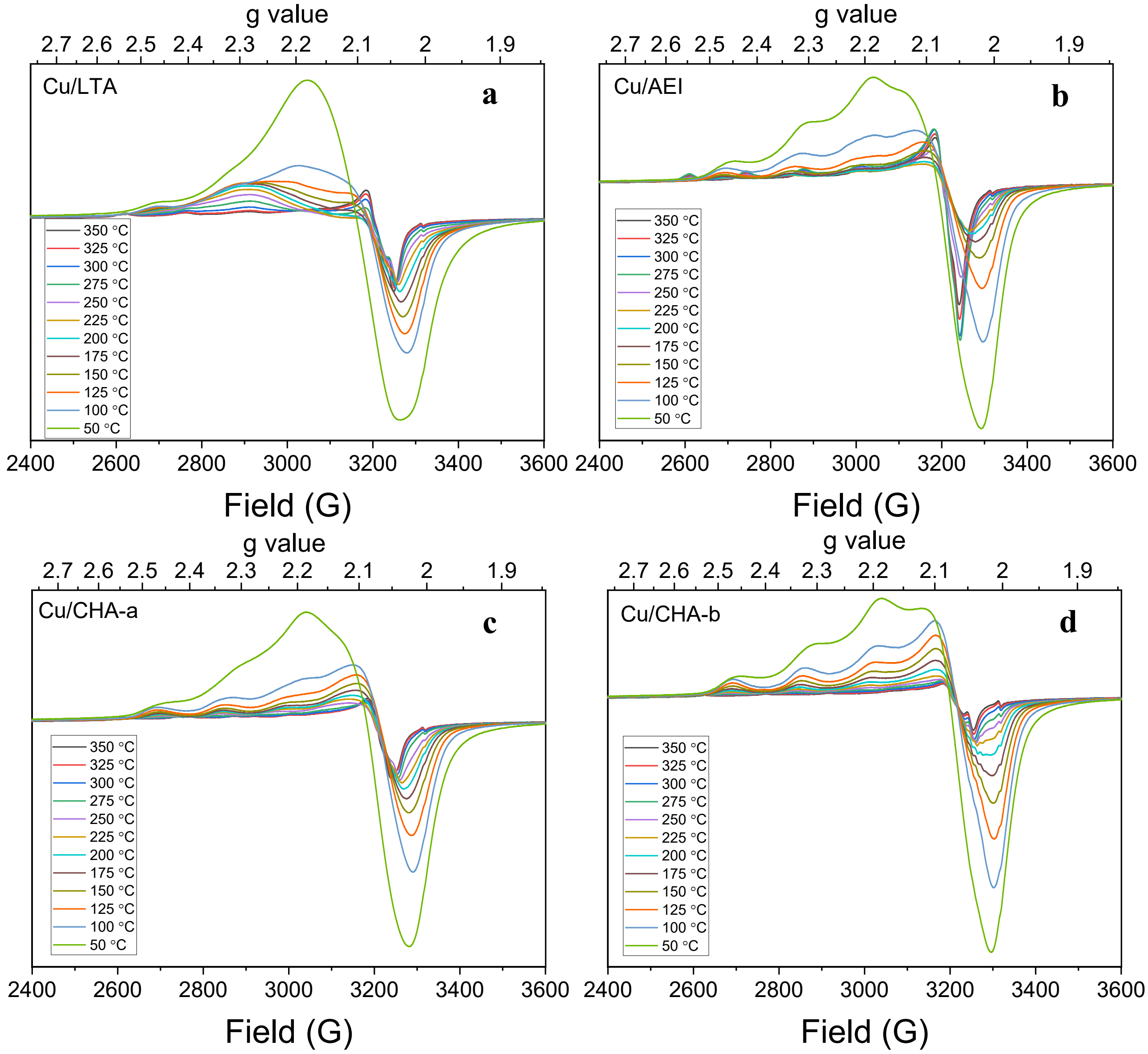

Supplementary Fig. 5 | *Operando* EPR spectra acquired during steady state standard SCR over the fresh catalysts. a, Cu/LTA. **b**, Cu/AEI. **c**, Cu/CHA-a. **d**, Cu/CHA-b. The reaction is carried out at temperatures from 100 to 350 °C at a GHSV of ~4×10^5 h^{-1}. The reactant feed contains 350 ppm NOx (containing ~10 ppm NO_2), 350 ppm NH_3, 10% O_2, and balanced N_2 at a GHSV of ~4×10^5 h^{-1}.

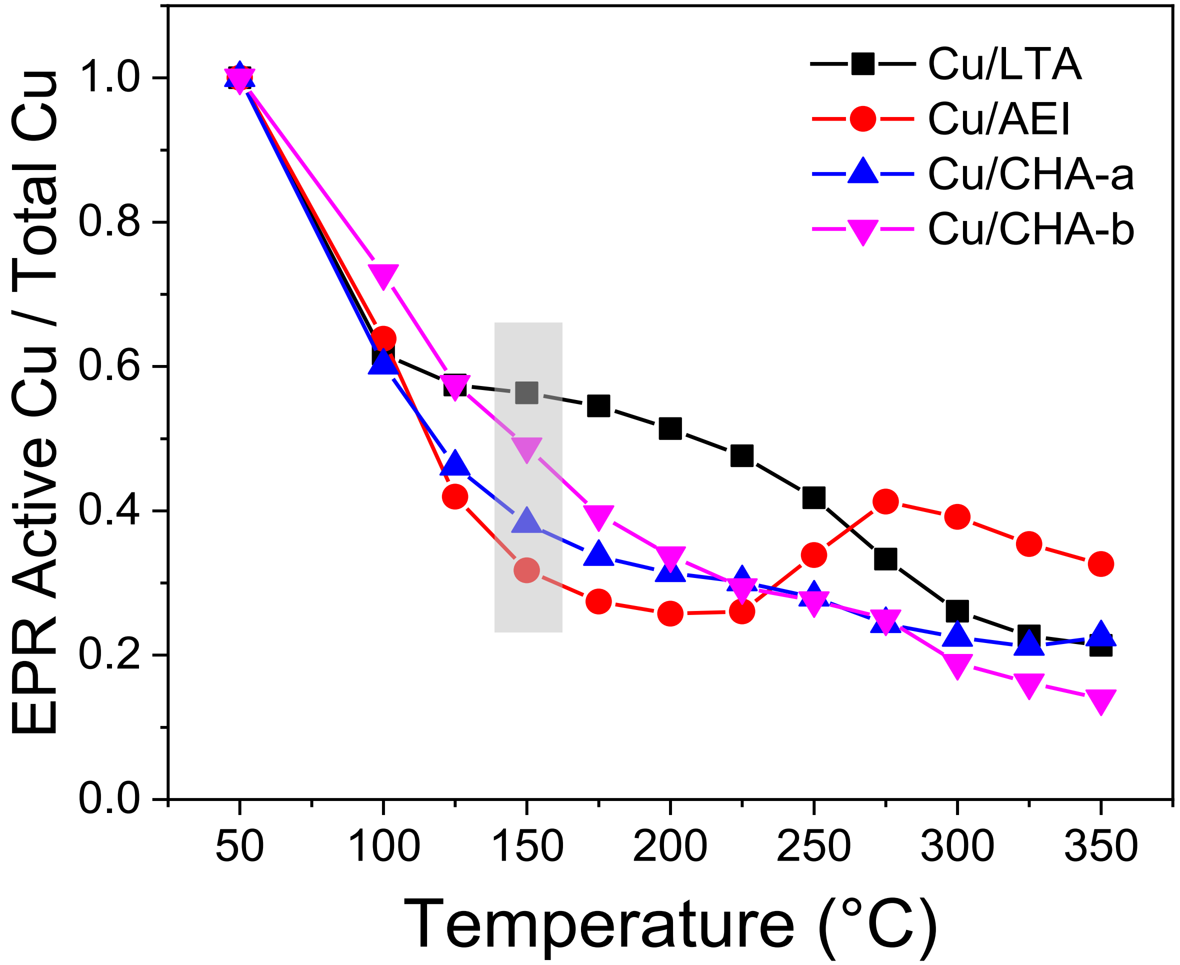

Supplementary Fig. 6 | Ratios between EPR-visible Cu^{II} and total Cu at various reaction temperatures during steady-state SCR over the fresh catalysts.

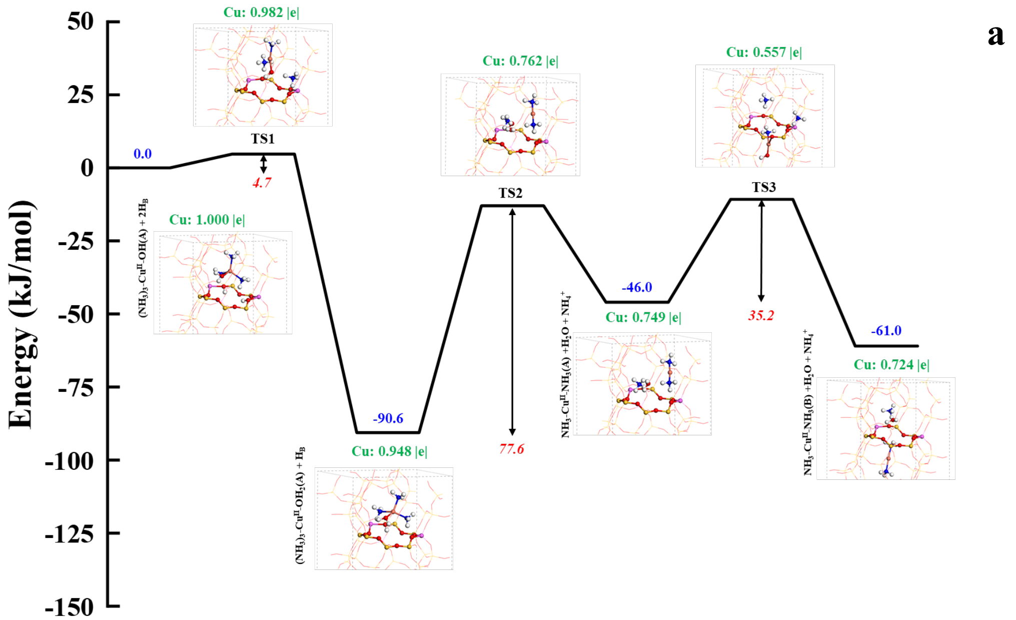

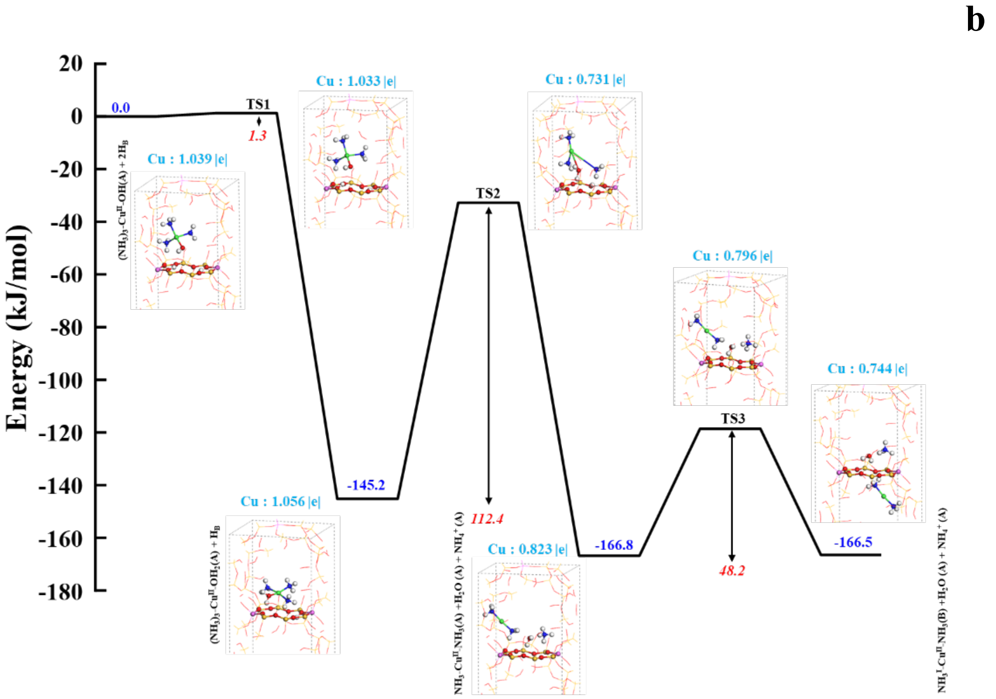

Supplementary Fig. 7 | Diffusion of $Cu^{II}(OH)(NH_3)_3$ intermediate through 8MR via the de-solvation path $Cu^{II}(OH)(NH_3)_3$ (A) + $2H_B^+ \rightarrow Cu^{II}(OH_2)(NH_3)_3$ (A) + $H_B^+ \rightarrow Cu^{II}(NH_3)_2$ (A) + H_2O + $NH_4^+ \rightarrow Cu^{II}(NH_3)_2$(B) + H_2O + NH_4^+. a, Cu/CHA. **b**, Cu/LTA.

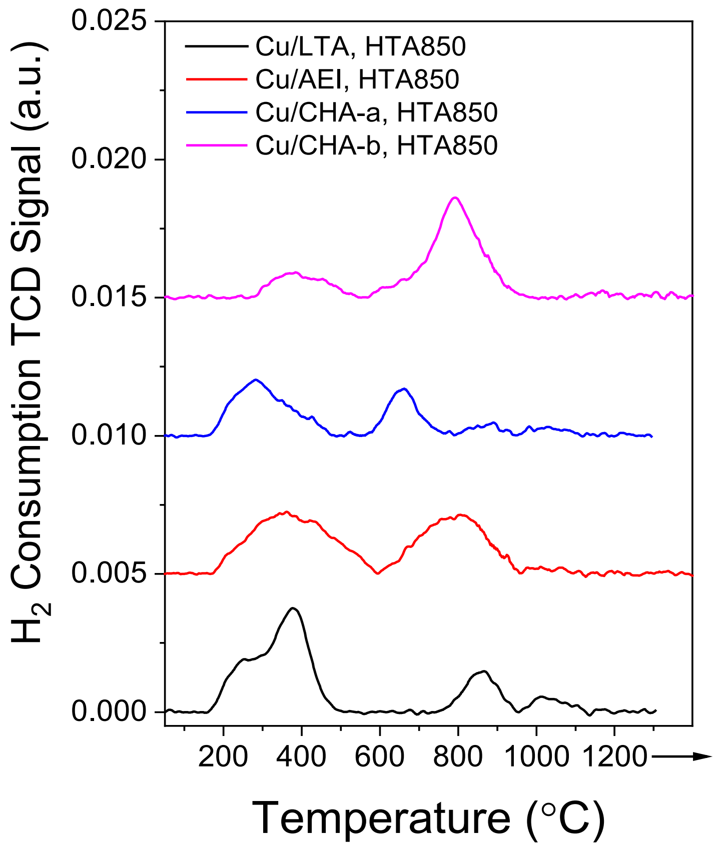

Supplementary Fig. 8 | H_2-TPR results for the 4 HTA850 catalysts. Reduction centered ~230 °C is due to $ZCu^{II}OH$ reduction to ZCu^{I}, reduction centered ~400 °C is due to Z_2Cu^{II} reduction to ZCu^{I}, and reduction above ~600 °C is due to ZCu^{I} reduction to metallic Cu[1, 2]. In comparison to H_2-TPR results for the fresh catalysts in Supplementary Fig. 3, two differences are obvious: (1) relative contents of Z_2Cu^{II} increase and relative contents of $ZCu^{II}OH$ decrease as a result of $ZCu^{II}OH + ZH \rightarrow Z_2Cu^{II} + H_2O$ that occurs during aging[6]. (2) the ZCu^{I} reduction shifts to lower temperatures, indicating the catalyst stability decrease caused by hydrothermal aging.

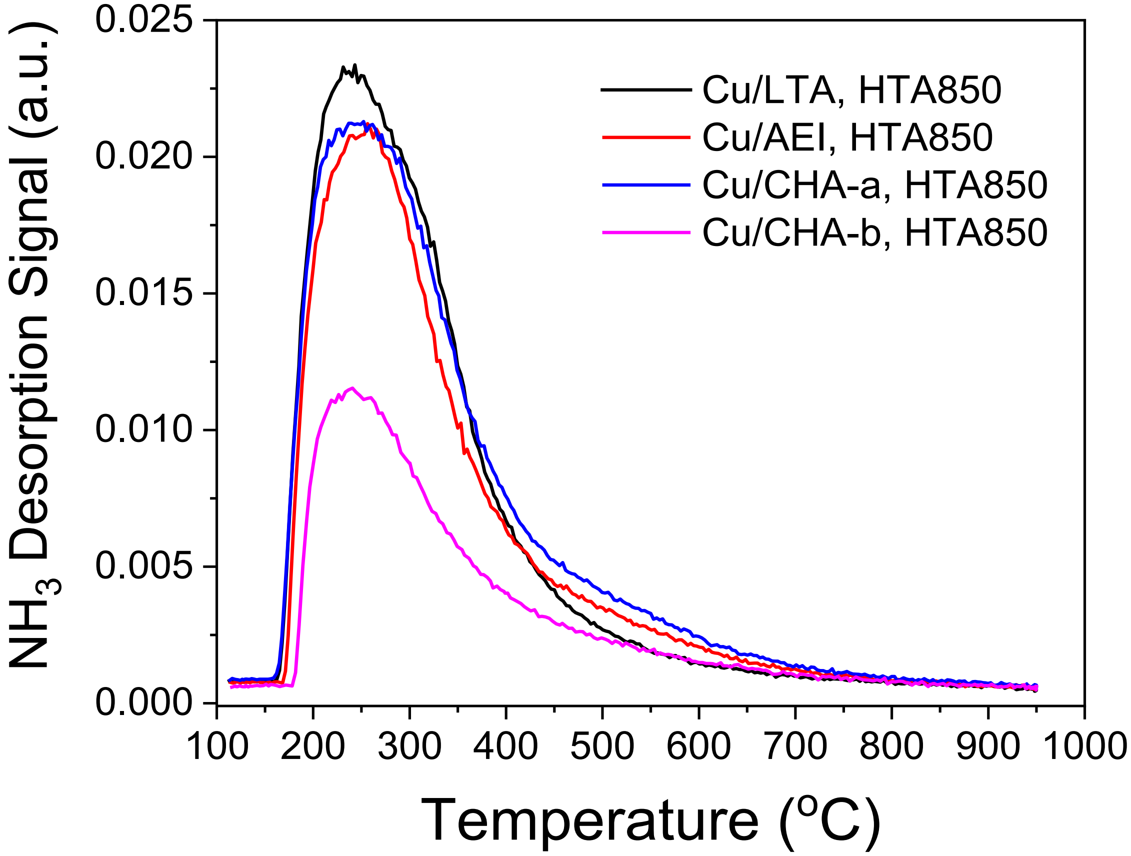

Supplementary Fig. 9 | NH_3-TPD results for the HTA850 catalysts. Desorption from weak acid sites, Cu-ions, and Brønsted acid sites is no longer well-resolved here.

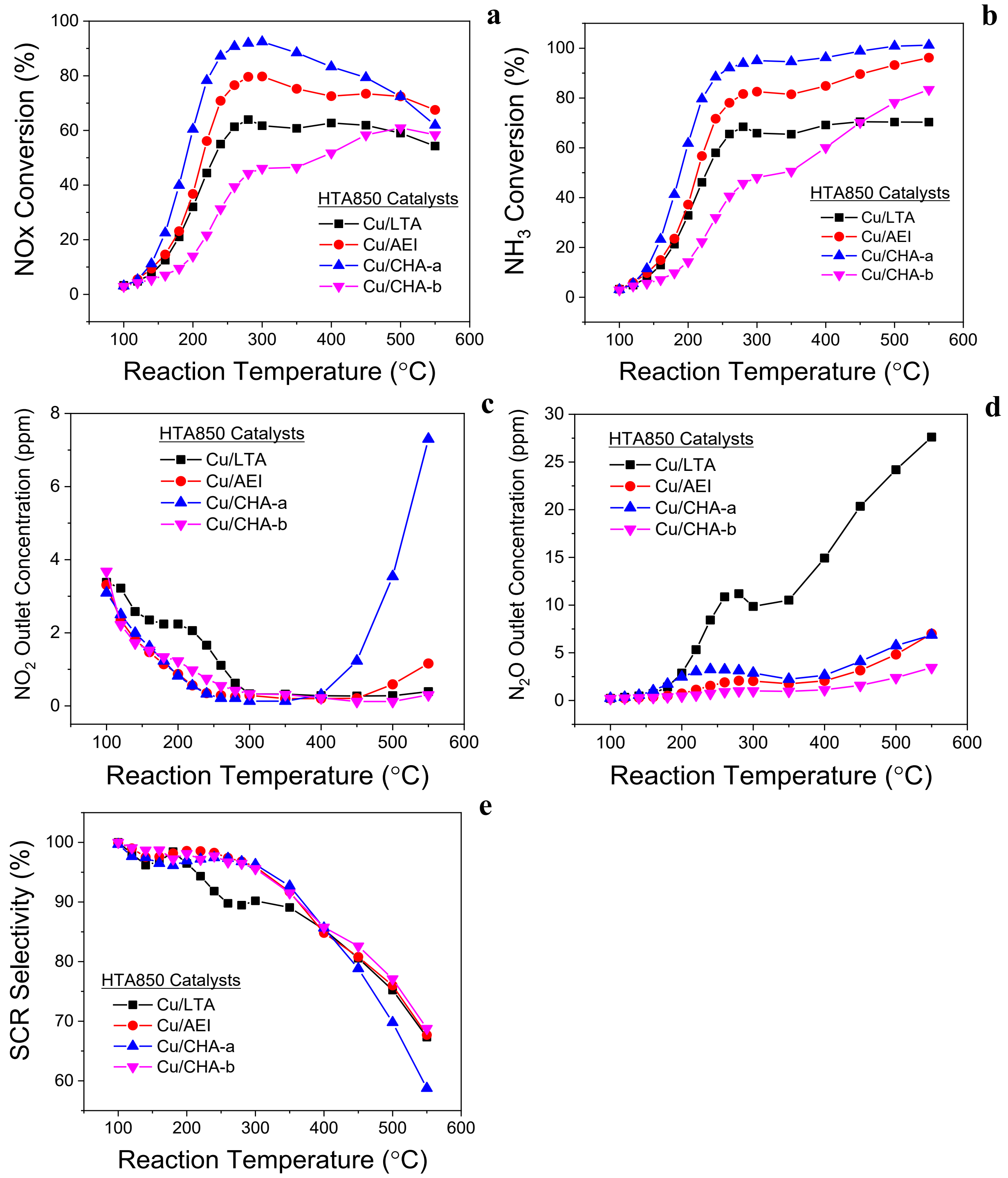

Supplementary Fig. 10 | Steady-state standard NH_3-SCR reaction test results on the 4 HTA850 catalysts. a, NOx conversion as a function of reaction temperature. **b**, NH_3 conversion as a function of reaction temperature. **c**, NO_2 outlet concentration (ppm) as a function of reaction temperature. **d**, N_2O outlet concentration (ppm) as a function of reaction temperature. **e**, SCR selectivity as a function of reaction temperature. SCR selectivity is calculated as: $\left(\frac{NO_x\ conversion}{NH_3\ conversion} -\right.$

$\frac{N_2O\ outlet\ concentration}{NO_x\ inlet\ concentration}$) ×100%. The reactant feed contains 350 ppm NOx (including ~10 ppm NO_2), 350 ppm NH_3, 2.5% H_2O, 10% O_2, and balanced N_2 at a gas hourly space velocity (GHSV) of ~2 × 10^5 h^{-1}.

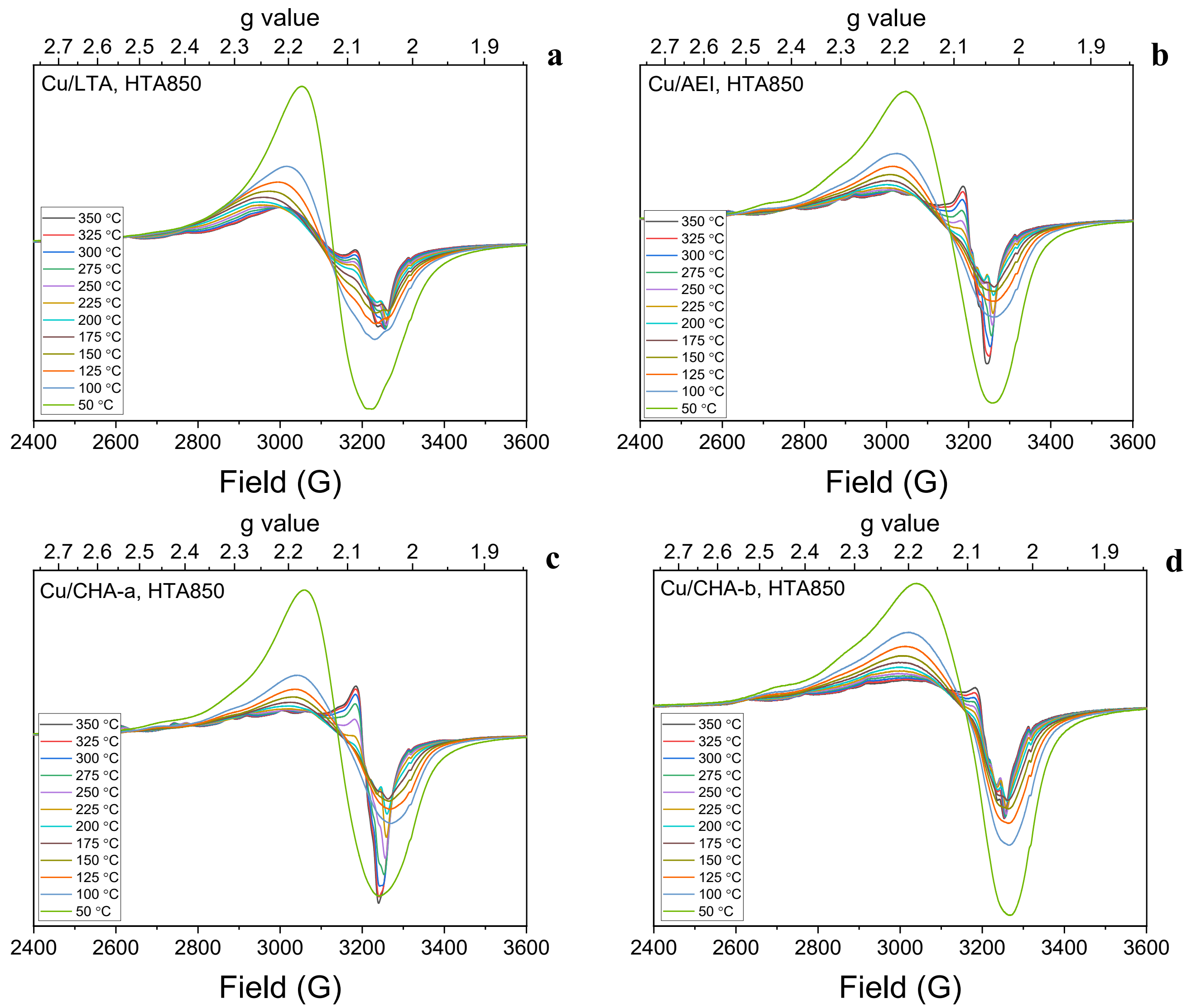

Supplementary Fig. 11 | *Operando* EPR spectra acquired during steady state standard SCR over the HTA850 catalysts. a, Cu/LTA, HTA850. **b**, Cu/AEI, HTA850. **c**, Cu/CHA-a, HTA850. **d**, Cu/CHA-b, HTA850. The reaction is carried out at temperatures from 100 to 350 °C at a GHSV of ~4×10^5 h^{-1}. The reactant feed contains 350 ppm NOx (containing ~10 ppm NO_2), 350 ppm NH_3, 10% O_2, and balanced N_2 at a GHSV of ~4 × 10^5 h^{-1}.

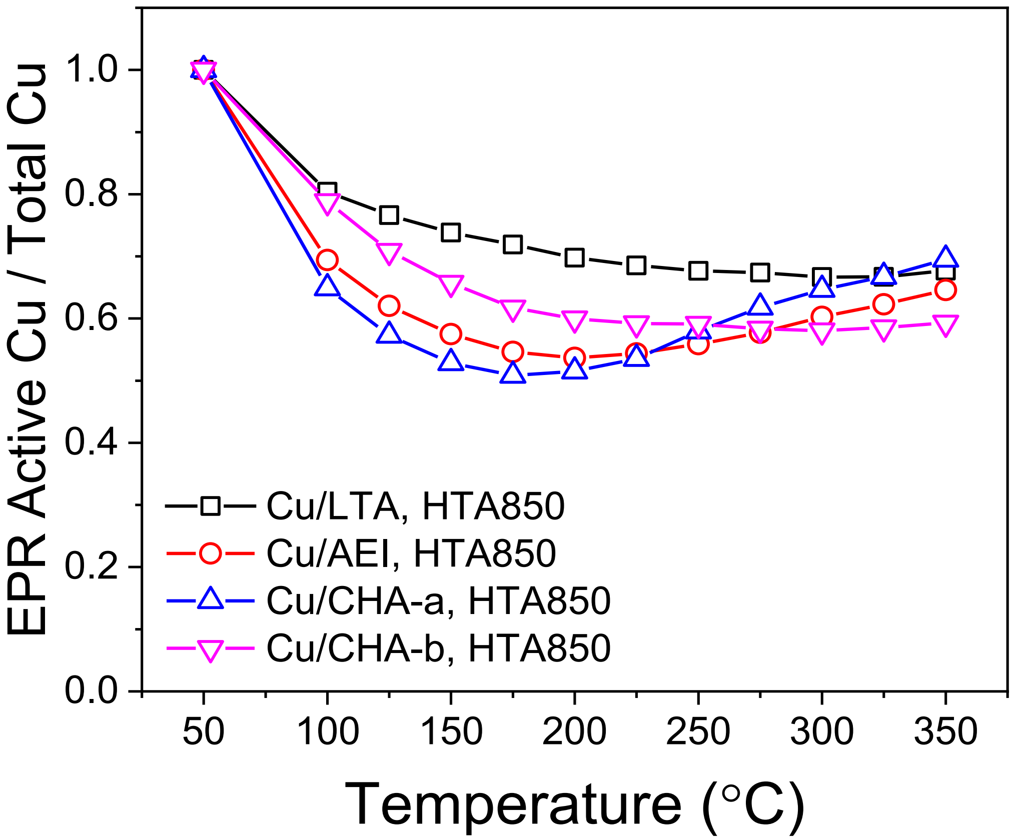

Supplementary Fig. 12 | Ratios between EPR-visible Cu^{II} and total Cu at various reaction temperatures during steady-state SCR over the HTA850 catalysts.

Supplementary Table 1 | Surface area/porosity results of the 4 zeolite supports measured by N_2 adsorption.

Sample	BET Surface Area (m^2/g)	Micropore Area (m^2/g)	External Surface Area (m^2/g)	t-plot Micropore Volume (cm^3/g)
LTA, fresh	703	641	62	0.248
AEI, fresh	556	498	58	0.189
CHA-a, fresh	729	667	62	0.251
CHA-b, fresh	774	717	57	0.269

Supplementary Table 2 | Useful textural and compositional properties of the 4 fresh catalysts. Si, Al and Cu contents of the catalysts were determined via ICP-AES; surface area/porosity results were measured by N_2 adsorption; both H_2-TPR and EPR were applied to obtain isolated Cu^{II}-ion content of the catalysts.

Catalyst Name	Si/Al	Cu loading (wt%, ICP)	BET Surface Area (m^2/g)	Micropore Volume (cm^3/g)	Isolated Cu(II) content (wt%, H_2-TPR)	Isolated Cu(II) content (wt%, EPR)
Cu/LTA	15.8	2.08	697	0.251	1.75	1.73
Cu/AEI	10.4	1.34	745	0.267	1.31	1.15
Cu/CHA-a	17.0	1.47	788	0.278	1.20	1.15
Cu/CHA-b	10.9	0.86	774	0.269	0.85	0.80

Supplementary Table 3 | Paired framework Al (-Al-Si-Al- or -Al-Si-Si-Al-) concentration estimated from Co-ion titration. Co contents were determined via ICP-AES. Titration results indicate that framework Al in AEI and CHA-a/b supports is roughly randomly distributed[7], however Al pairing is enriched in LTA.

Support	Si/Al	Co loading (wt%, ICP)	Co/Al
LTA	15.8	1.98	0.39
AEI	10.4	1.74	0.24
CHA-a	17.0	1.18	0.25
CHA-b	10.9	2.10	0.27

Supplementary Table 4 | Useful textural and compositional properties of the 4 HTA850 catalysts. Si, Al and Cu contents of the catalysts were determined via ICP-AES; surface area/porosity results were measured by N_2 adsorption; EPR was applied to obtain isolated Cu^{II}-ion content of the catalysts.

Catalyst Name	Si/Al	Cu loading (wt%, ICP)	BET Surface Area (m^2/g)	Micropore Volume (cm^3/g)	Isolated Cu(II) content (wt%, EPR)
Cu/LTA, HTA850	15.8	2.08	638	0.205	0.95
Cu/AEI, HTA850	10.4	1.34	721	0.266	0.87
Cu/CHA-a, HTA850	17.0	1.47	771	0.270	0.87
Cu/CHA-b, HTA850	10.9	0.86	755	0.256	0.58

Supplementary Table 5 | Relative intensities of Al^{Td}, Al^{P} and Al^{Oh} signals derived by peak fitting of the ^{27}Al NMR spectra shown in Fig. 5 of the main text. By assuming that the higher the preservation of Al4 sites, the better the hydrothermal stability, it is estimated that hydrothermal stability of the catalysts follows the order Cu/LTA > Cu/CHA-a > Cu/AEI > Cu/CHA-b.

Catalyst Name	Al^{Td} (%)	Al^{P} (%)	Al^{Oh} (%)
Cu/LTA, HTA850	79	5	16
Cu/AEI, HTA850	56	24	20
Cu/CHA-a, HTA850	68	12	20
Cu/CHA-b, HTA850	51	27	22

Supplementary References:

1. Gao, F. et al. Structure-activity relationships in NH3-SCR over Cu-SSZ-13 as probed by reaction kinetics and EPR studies. *J Catal* **300**, 20-29 (2013).
2. Cui, Y.R. et al. Revisiting effects of alkali metal and alkaline earth co-cation additives to Cu/SSZ-13 selective catalytic reduction catalysts. *J Catal* **378**, 363-375 (2019).
3. Andersen, C.W. et al. Location of Cu^{2+} in CHA zeolite investigated by X-ray diffraction using the Rietveld/maximum entropy method. *IUCrJ* **1**, 382-386 (2014).
4. Zhang, Y.N. et al. Quantitative Cu Counting Methodologies for Cu/SSZ-13 Selective Catalytic Reduction Catalysts by Electron Paramagnetic Resonance Spectroscopy. *J Phys Chem C* **124**, 28061-28073 (2020).
5. Luo, J.Y. et al. New insights into Cu/SSZ-13 SCR catalyst acidity. Part I: Nature of acidic sites probed by NH3 titration. *J Catal* **348**, 291-299 (2017).
6. Zhang, Y.N. et al. Probing Active-Site Relocation in Cu/SSZ-13 SCR Catalysts during Hydrothermal Aging by In Situ EPR Spectroscopy, Kinetics Studies, and DFT Calculations. *Acs Catal* **10**, 9410-9419 (2020).
7. Paolucci, C. et al. Catalysis in a Cage: Condition-Dependent Speciation and Dynamics of Exchanged Cu Cations in SSZ-13 Zeolites. *J Am Chem Soc* **138**, 6028-6048 (2016).
